# Potential blue carbon in the fringe of Southern European Kelp forests

**DOI:** 10.1038/s41598-025-09361-9

**Published:** 2025-08-12

**Authors:** João N. Franco, Hugo Sainz Meyer, Óscar Babe, Marta Martins, Bianca Reis, Álvaro Sanchez-Gallego, Marco F. L. Lemos, Marina Dolbeth, Isabel Sousa-Pinto, Francisco Arenas

**Affiliations:** 1 MARE—Marine and Environmental Sciences Centre & ARNET—Aquatic Research Network Associated Laboratory, ESTM, Polytechnic of Leiria, Peniche, Portugal; 2 CIIMAR—Interdisciplinary Centre of Marine and Environmental Research, University of Porto, Matosinhos, Portugal; 3 Department of Biology, Faculty of Sciences, University of Porto, Rua do Campo Alegre 1021 1055, Porto, 4169-007 Portugal

**Keywords:** Carbon sequestration, *Laminaria hyperborea*, *Saccorhiza polyschides*, Portugal, Seaweed forests, Ecology, Ecology

## Abstract

Blue Carbon encompasses the organic carbon sequestered and stored by coastal and marine ecosystems, including seaweed forests. This study aims to quantify the potential Blue Carbon storage and sequestration rates of subtidal kelp forests in Northern Portugal, focusing on the most dominant species *Laminaria hyperborea* and *Saccorhiza polyschides*. Through in-situ measurements of forest extension, biomass, growth, and carbon content, we determined that these kelp forests store approximately 16.48 Gg of carbon in aboveground biomass, covering an area of 5189 hectares. The estimated carbon sequestration rate is 1903 Mg C year^–1^, which is lower than the 3717 Mg C year^–1^ sequestered by saltmarshes and seagrasses in mainland Portugal (available data). However, when normalised by area, kelp forests sequester carbon at rates that equal or exceed those of saltmarshes and seagrasses, underscoring their significance for regional carbon management. Despite this, export assumptions significantly influence total carbon flux estimates, and large portions of the Portuguese coastline remain understudied. Expanding in-situ assessments, combined with regional modelling would improve Blue Carbon estimates and lay the groundwork for a future national-scale assessment. Given their potential role in climate mitigation, conservation and restoration efforts should be prioritized to protect and enhance the Blue Carbon capacity of kelp forests and associated habitats.

**Supplementary Information:**

The online version contains supplementary material available at 10.1038/s41598-025-09361-9.

## Introduction

Blue Carbon (BC) encompasses the organic carbon stored by coastal as biomass in marine ecosystems, with a particular emphasis on coastal vegetated areas such as saltmarshes, mangrove forests, and seagrass meadows^1,2^. The escalating impact of climate change requires accurate identification and quantification of BC, as it can aid mitigating current emissions^1,3,4^. Coastal vegetated ecosystems—mangroves, saltmarshes and seagrass meadows—have long been central to blue-carbon budgets because their plant biomass promotes sediment accumulation and long-term carbon burial, whereas non-accreting marine habitats such as macroalgal forests have received far less attention^5^. Despite its importance, BC has been largely excluded from formal carbon-offset frameworks, because of persisting uncertainties over sequestrating permanence, the lack of standard monitoring and verification protocols, and unresolved governance and financing issues^3,6,7^. As a result, some of the most efficient natural habitats for carbon sequestration and storage—along with their associated ecosystem services benefits - are mostly overlooked^8,9^; however, national-scale voluntary schemes such as Japan’s J-Blue Credit programme—which already certifies blue-carbon offsets from eelgrass and kelp projects—show how these ecosystems can be integrated into carbon markets^6,10^. While terrestrial forests are already supported by well-established carbon-off set mechanisms such as REDD (Reducing emissions from Deforestation and forest Degradation), the carbon-sequestration potential of marine vegetated ecosystems - including seaweed forests - remains underexplored^11^. Integrating BC into carbon offsetting frameworks requires overcoming scientific challenges, such as resolving controversies around the inclusion of macroalgae and the role of carbonate cycling in BC accounting, as well as policy and governance challenges, including technical and financial barriers and equity and justice issues^3,11,12^. Indeed, recent studies have increasingly focused on the inclusion of other neglected components within marine systems, such as non-accreting habitats like seaweed forests, highlighting their importance as integral parts of BC budgets^1,11–15^.

Kelp forests, which dominate around 26% of global coastlines, are extensive habitats with substantial biomass production and detritus export potential^14,16^. Their net primary production per unit area is among the highest of all other habitats in the world^15^. Traditionally considered poor in carbon burial, recent evidence challenges this notion by revealing their contribution to allochthonous biomass in other ecosystems^17^. Indeed, recent estimations byFilbee-Dexter et al. (2024) indicate that, on average, 16% of global seaweed production, including kelp, is exported across the continental shelf (≈ 55 Tg C yr⁻¹) and thereby accounts for long-term carbon burial, while kelp forests simultaneously provide habitat for diverse marine communities and underpin coastal food webs^16^. Despite their ecological significance, kelp forests in Europe, particularly those along the southern Atlantic coast, remain understudied and therefore their BC potential is unknown. The subtidal of the western coast of Portugal spans approximately 700 km, extending from Caminha to Sagres, encompassing areas dominated by rocky reefs or sand. Historically, rocky reefs in the northern, central, and southern regions have been occupied by kelp forests^18^. However, over the past decade, persistent kelp beds have been observed primarily in the northern and central regions, while the southern reefs have been dominated by turf and foliose algae^19–21^. These forests, despite being in decline^19,20,22,23^ maintain considerable standing biomass, particularly in the subtidal reefs of northern region dominated by the kelp species *Laminaria hyperborea* and *Saccorhiza polyschides*^19,21^.

Addressing this knowledge gap regarding blue carbon (BC) stocks and fluxes in Portuguese kelp forests holds significant potential to inform and enhance conservation, restoration, and management strategies for kelp ecosystems in southern Europe. This information could provide a scientific foundation for evidence-based policies aimed at protecting these vital marine habitats. Northern Portugal is an interesting case study for such quantification, given its seaweed composition and oceanographic conditions closely resemble those of the northern Atlantic coasts. This region also offers an opportunity to better understand the variability in regional blue carbon potential. Our research aims to fill this gap by quantifying blue carbon in this unexplored region. Thus, the objective of this study was to assess the potential blue carbon of the northern Portugal kelp forests, focusing on the dominant kelp species. Using in *situ* collected data, such as bottom substrate, kelp abundance, kelp growth, and kelp carbon content, we have determined the overall blue carbon potential in this region.

## Materials and methods

**Fig. 1:**
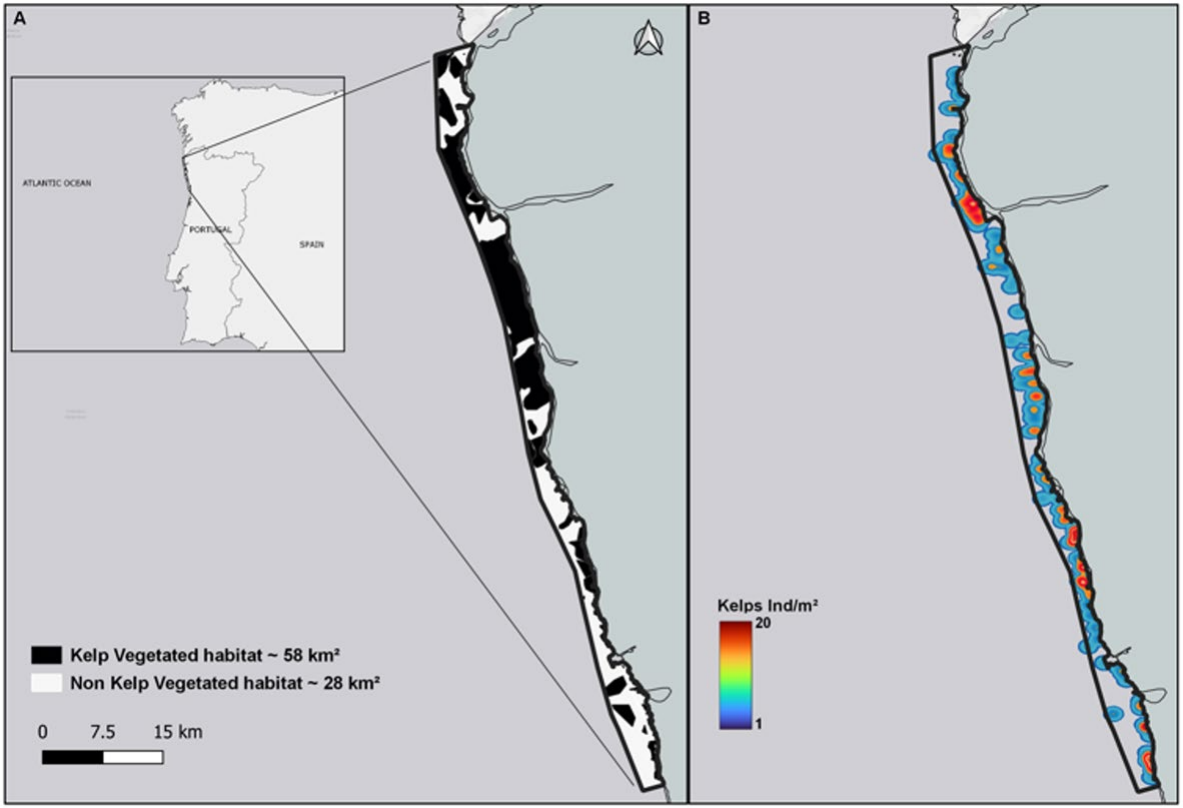
Spatial distribution of kelp forests along the Northern Portuguese coast. (A) represents the study area, showing kelp-vegetated habitats (black) and non-vegetated habitats (white). (B) illustrates kelp density (individuals/m^2^) using a heatmap representation.

The blue carbon assessment of Northern Portugal was based on observation and in-situ measurements. We examined the extension of kelp forests across a 90 km stretch of coastline (Fig. 1A, B), verifying seabed substrate and estimating the proportion of rocky reefs occupied by different kelp species. We conducted 496 video camera (QYSEA FIFISH PRO) point surveys (along virtual lines perpendicular to the coast) to estimate the suitable rocky substrate occupied by kelp, specifically *Saccorhiza polyschides* and *Laminaria hyperborea*, the dominant species of the subtidal reefs in northern Portugal^19^. We determined the total available substrate by combining surface sediments data from the GIS layer of the Portuguese Hydrographic Institute with video camera surveys and adopting a conservative depth limit of 20 m, although kelps can be found at depths greater than 50 m^24–26^.

Carbon stock and sequestration were estimated by adapting the methodology of Filbee-Dexter and Wernberg (2020). We calculated total kelp abundance by multiplying the reef area occupied by kelp with the average abundance ± standard deviation of the dominant species, *Saccorhiza polyschides* and *Laminaria hyperborea*. We used collected video image where kelps occurred (*n* = 303), classified on an SACFOR scale (Superabundant, Abundant, Common, Frequent, Occasional, Rare) and translated to corresponding abundances^23,27^. When present, new recruits (< 15 cm) were excluded due to identification and quantification difficulties, and their biomass is minimal compared to that of adult individuals^28^. Biomass data were obtained from 179 samples (113 *L. hyperborea*, 66 *S. polyschides*), primary productivity was calculated from 119 individual growth rates (72 *L. hyperborea*, 47 *S. polyschides*), using the punch-hole method^29^ and carbon content in kelp tissue was determined from 36 samples (18 *S. polyschides*, 18 *L. ochroleuca*) in the same regions. For the carbon content, random individuals were collected, dried at 60 °C for 48 h, and crushed to a fine powder for analysis. Total carbon content was measured using an Organic Elemental Analyzer – Flash 2000 (Thermo Fisher Scientific, Milan, Italy) and expressed as a percentage of dry weight. Carbon production rates were calculated using the average net primary production measured in 119 kelp individuals (72 *L. hyperborea* and 47 *S. polyschides*) and the percentage of seaweed net primary production (NPP) that is sequestered to deep ocean based on estimates Krause - Jensen and Duarte (2016) − 11% (range: 4–18%). To avoid double counting kelp carbon, we conservatively used a sequestration rate of 10.1% NPP, excluding continental shelf burial. Only 0.9% of NPP is buried on the continental shelf, with even less reaching saltmarshes, mangroves, and seagrass beds^30^. We compared the contribution of kelp forests to other blue carbon habitats in Portugal using the most recent and available data from the Blue Carbon Portugal report^31^. Source data and calculations are provided as a Supplementary Data file.

## Results and discussion

### Aboveground biomass and blue carbon storage

We calculated that the Northern Portugal kelp forests store an aboveground biomass of approximately 16.48 Gg C, accounting for 14,5% of the total aboveground biomass of blue carbon in the continental habitats (Table 1). The remaining 85.5% of the stock is distributed among saltmarshes (83.5%) and seagrasses (2.0%), as reported by Santos et al. (2023). In these two habitats, only 2% of carbon is stored in the biomass in seagrasses, while in saltmarshes, this value increase to 13%. Accordingly, these values were considered as above-ground biomass in seagrasses and saltmarshes, respectively, and they represent estimates for the entire continental Portugal^31^. Among the dominant kelp species, *Saccorhiza polyschides* contributes the most, with a stock biomass of 15.47 Gg C, while *Laminaria hyperborea* accounts for 1.02 Gg C. Comparable, site-specific biomass estimates for seagrass and saltmarsh habitats are not yet available for northern Portugal; consequently, we use the national values of Santos et al. (2023) as the best current proxy and highlight the need for dedicated regional surveys. The total area of these kelp forests is 5189 hectares (ha) in northern Portugal alone, making them a significant component of the blue carbon habitat in this region. The aboveground biomass per unit area for the dominant kelp species, *Laminaria hyperborea* and *Saccorhiza polyschides*, was found to be 0.52 Mg C ha^–1^ ± 0.29 Mg C ha^–1^ and 4.77 Mg C ha^–1^ ± 1.97 Mg C ha^–1^ respectively. Combined, these kelp species contribute to a mean biomass of 2.65 Mg C ha^–1^ ± 1.19 Mg C ha^–1^ (Table 1). These values indicate that the kelp forests in Northern Portugal have a substantial biomass, which is crucial for carbon absorption, with the potential to contribute to long-term carbon storage.

**Table 1:** Estimated aboveground biomass and blue carbon stock of dominant Kelp species (*Laminaria hyperborea* and *Saccorhiza polyschides*) in Northern portugal.

Dominant Kelp species northern Portugal	Aboveground biomass (Mg C ha-1)		Total area (ha)		Stock above ground biomass (GgC)
	Mean	SD			
*Laminaria hyperborea*	0,52	0,29	1946		1,02
*Saccorhiza polyschides*	4,77	1,97	3243		15,47
Both kelp species	2,65	1,19	5189	Total	16,48

Values represent mean aboveground biomass (Mg C ha^–1^) with standard deviation (SD), total area (ha) covered by each species, and the estimated stock of aboveground biomass (GgC).

### Blue carbon sequestration

The sequestration rate for the kelp forests in Northern Portugal is estimated to be 0.60 ± 0.05 Mg C ha^–1^ year^–1^, leading to an estimated total sequestration of 1903 Mg C year^–1^ across the 5189 ha area (Tables 2 and 3). This represents 33.9% of the total annual blue carbon sequestration in continental Portugal. In comparison, saltmarshes sequester 2930 Mg C year^–1^ and seagrasses contribute 787 Mg C year⁻¹^31^. These values correspond to 52.1% and 14.0% of the total sequestration, respectively, as calculated in this study. Kelp forests in Northern Portugal exhibit a considerable total sequestration rate despite covering a smaller area compared to saltmarshes (Table 3). The aboveground carbon stock and sequestration rates of these kelp forests underscore their significant role in the regional carbon budget. When considering their combined area and biomass, these northern kelp forests make a notable contribution to the overall blue carbon sequestration in Portugal.

**Table 2:** Estimated carbon sequestration rates of dominant Kelp species (*Laminaria hyperborea* and *Saccorhiza polyschides*) in Northern portugal.

Dominant Kelp species northern Portugal	Sequestration rates (Mg C ha-1 year-1)		Total area (ha)		Sequestration rates (Mg C year-1)
	Mean	SD			
*Laminaria hyperborea*	0,49	0,02	1946		952
*Saccorhiza polyschides*	1,28	0,09	3243		4138
Both kelp species	0,88	0,05		Total	5090

The table presents mean sequestration rates (Mg C ha^–1^ year^–1^) with standard deviation (SD), total habitat area (ha), and the total estimated carbon sequestration per year (Mg C year^–1^) for each species and their combined contribution.

**Table 3:** Carbon sequestration rates across blue carbon habitats in continental portugal, including saltmarshes, seagrasses, and Kelp forests.

Blue carbon habitats	Sequestration rates (Mg C ha-1 year-1)	Total area (Mha)	Sequestration rates (Mg C year-1)	% of Sequestration rate in continental Portugal	Source
Tidal Marsh (continental Portugal)		10,039	2930	52,1%	Santos et al., 2023
Seagrass (continental Portugal)		1684	787	14,0%	Santos et al., 2023
Kelp forest (northern Portugal)	0,60 ± 0,05	5189	1903	33,9%	This study
Total		16,912	5620	100%	

The table presents mean sequestration rates (Mg C ha^-1^ year^-1^), total habitat area (Mha), total estimated carbon sequestration per year (Mg C year^-1^), and the percentage contribution of each habitat to the total blue carbon sequestration in continental portugal. Data for saltmarshes and seagrasses are sourced from Santos et al. (2023), while Kelp forest values are from this study.

### Comparison with traditional blue carbon ecosystems

The total surface area of kelp forests in Northern Portugal (5189 ha) is smaller compared to the cumulated autochthonous habitats i.e. saltmarshes (10039 ha) and seagrasses (1684 ha) in continental Portugal (Tables 3 and 4). However, the aboveground biomass of these kelp forests (16.48 Gg C) is ~ 7 times higher than that of seagrasses (2,26 Gg C), and only 5.8 times lower than that of saltmarshes (95.16 Gg C). This suggests that, despite their smaller extent compared to saltmarshes, kelp forests contribute a significant proportion of aboveground biomass. It is important to note that our calculations only considered the aboveground biomass of seagrasses and saltmarshes, which represent just 2% and 13% of their total ecosystem carbon stock, respectively^31^. Therefore, direct comparisons with kelp forests should be interpreted with caution, as a substantial portion of the carbon stock in seagrasses and saltmarshes is stored in belowground biomass and sediments. Additionally, despite being the most recent and comprehensive data compilation of Blue Carbon provided by seagrasses and saltmarshes in mainland Portugal^31^, acknowledges that the data is incomplete and outdated. The study also lacks coverage of significant Northern Portugal systems such as the Minho or Lima estuaries, which host extensive salt-marsh and seagrass meadows and therefore likely contain substantial ecosystem carbon stock, but have not yet been mapped in detail because long-term benthic-vegetation monitoring programmes and high-resolution remote-sensing data are still lacking for these sites (Santos et al., 2023).

**Table 4:** Summary of biomass carbon stocks in coastal habitats of continental portugal, including saltmarshes, seagrasses, and Kelp forests.

Blue carbon habitats	Biomass (Mg C ha^− 1^)	Total area (ha)	Stock biomass (GgC)	% of Total biomass carbon stock in Continental Portugal	Source
Tidal Marsh (continental Portugal) ^a^		10,039	95,16	83,5%	Santos et al. 2023
Seagrass (continental Portugal) ^a^		1684	2,26	2,0%	Santos et al. 2023^**b**^
Kelp forest (northern Portugal)	2.65 ± 1,19	5189	16,48	14,5%	This study
Total		16,912	113,90	100,0%	

Biomass (Mg C ha^–1^ ± SD) where available, total habitat area (ha), estimated stock biomass (GgC), and the percentage contribution of each habitat to the total aboveground blue carbon stock in continental portugal. Data for saltmarshes and seagrasses are sourced from^31^while Kelp forest values are from this study.

^a^ In seagrasses, only 2% of the carbon is stored in the biomass, while in salt marshes, this value increases to 13%. Therefore, these values were considered as above-ground biomass in seagrasses and saltmarshes, respectively.

The total sequestration rates for saltmarshes and seagrasses in mainland Portugal amounts to 3717 Mg C year^–1^, while kelp forests in Northern Portugal contribute an estimated 1903 Mg C year^–1^. However, these values should be interpreted with caution, as export assumptions significantly influence total carbon flux estimates. In this study, we assumed a biomass export rate of 10.1%^12,27^, whereas Filbee-Dexter et al. (2024) modelled a higher export rate of 36.7% for seaweeds in Portugal. These values, like those in all other studies, are not absolute, as accurate estimations in marine environments are inherently challenging due to biological, environmental, and hydrodynamic variability^9,32,33^. Nevertheless, this comparison highlights the important role of kelp forests in regional carbon sequestration strategies, supporting previous findings on their significance contribution to blue carbon budgets^1,5,11,12^. Given their potential, conservation and restoration efforts should be prioritized to maximize their carbon sequestration capacity.

This study complements the work of Filbee-Dexter et al. (2024) by providing fine-scale, in-situ data that align with and reinforce the broader patterns identified through their national-scale modelling approach. Despite differences in methodology and spatial scale, both studies reveal valuable insights and proportionalities, reinforcing the importance of kelp forests as key blue carbon habitats.

This study, based on in situ data, examined a specific area of the Northern Portuguese coast, covering 80 km^2^ with a vegetated area of 51,89 Km^2^ (5189 ha), and reported an aboveground biomass of 16.48 Gg C, assuming 10.1% of net primary production (NPP) is exported. This results in a sequestration rate of 600 Mg C year^–1^ m^–2^ and a total exported biomass of 310 Mg C year^–1^. In contrast, Filbee-Dexter et al. (2024) modelled the entire Portuguese coast (1674 km^2^), estimating a total of vegetated area of 1674 Km2, an aboveground biomass of 924.4 g C year^–1^ m^–2^ and an export rate of 35,6%. These assumptions resulted in an exported biomass per area of 280.9 g C year^–1^ m^–2^ and a total exported biomass of 0.47 Tg C year^–1^ (range: 0.17–1.10 Tg C year^–1^).

Although our estimated export is ≈ 6 g C m^–2^ yr^–1^ (0.06 Mg C ha^–1^ year^–1^) it remains an order of magnitude lower than the ≈ 281 g C m^–2^ yr^–1^ ( 2.81 Mg C ha^–1^ year^–1^) predicted by Filbee-Dexter et al. (2024), it is essential to consider the differences in study area and taxa covered. Our study only includes Laminariales and Tilopteridales and represents only ~ 3% of the vegetated area reported in Filbee-Dexter et al. (2024). Their study also accounts for Fucales and Desmarestiales, which may contribute significantly to the total carbon stock. When adjusting our assumptions to align with Filbee-Dexter et al. (2024)’s export rate (36.7%), our total exported biomass increases to 0.00114 Tg C year^–1^, bringing our estimates closer to the range reported by their study. On the other hand, if we apply our 10.1% export assumption to Filbee-Dexter et al. (2024) model, their exported biomass will decrease to 0.122 Tg C year^–1^, reducing the gap between the two studies.

These findings suggest that if standardization per unit of area were applied across Portugal, the final total exported biomass estimations (Tg C year^–1^) might not be as different as initially perceived. Instead of conflicting results, the two studies provide complementary insights, highlighting the importance of both in situ measurements and large-scale modelling approaches in refining blue carbon assessments.

This alignment suggests that, despite methodological differences, the localized data from this study are consistent with broader trends observed in the Filbee-Dexter et al. (2024) study. The higher biomass density reported in this study could be due to the specific characteristics of the Northern Portuguese coast, which may have more favourable conditions for kelp growth^19,21,34,35^. It should be noted that the remaining coast of Portugal still lacks detailed studies like the present one. While absolute values differ, the proportions indicate that the localized records of this study are within a reasonable range of broader estimates. Our export intensity (≈ 6 g C m^–2^ yr^–1^; 0.06 Mg C ha^–1^ yr^–1^) is similar to values modelled for the United Kingdom (≈ 16 g C m^–2^ yr^–1^ and well below the upper range reported for other temperate coasts such as Spain (≈ 195 g C m^–2^ yr^–1^; Filbee-Dexter et al., 2024), indicating that the Portuguese estimates lie within the broader global spectrum. This underscores the importance of integrating localized in situ data with large-scale modelling approaches to enhance the accuracy and relevance of blue carbon assessments. Future efforts should refine the key export assumptions—namely (i) the fraction of kelp net primary production that is transported beyond the shelf and (ii) the share that becomes permanently buried in continental-shelf and deep-coastal sediments—while improving regional estimations and expanding in situ monitoring to strengthen our understanding of Portuguese kelp forests as blue carbon ecosystems.

### Future research directions

To improve the accuracy of blue carbon assessments, more detailed data on the extent, production, and sequestration rates of seaweed forests, as well as refining decomposition rates, carbon sink estimates, and long-term sequestration potential, are essential where also geomorphological factors like distance to coast and slope are key variables^11,32^. These efforts will help reduce uncertainties and highlight the complexity of studying marine carbon cycles, emphasizing the need for ongoing research in this field. Enhanced carbon export models, such as those proposed by Krause-Jensen and Duarte (2016) and Filbee-Dexter et al. (2024), along with estimates of dissolved organic carbon (DOC) fluxes and shelf burial^17,36,37^ will contribute to further refining these assessments. An improved understanding of the proportion of net primary production that is sequestered through burial in deep ocean sediments or transported below the mixed layer is necessary.

The importance of in situ and regional sampling cannot be overstated. The growth of kelp species fluctuates throughout the year, and the rocky substrate is not uniformly occupied by different kelp species. For instance, *Saccorhiza polyschides* and *Laminaria hyperborea* occupy different depths and niches, with annual and perennial species shedding different amounts of biomass. Moreover, coralline algae, often overlooked in blue carbon assessments, are highly productive and contribute significantly to the oceanic carbon cycle. In addition to kelps, coralline algal may also contribute to the oceanic carbon cycle through both organic carbon fixation and long-term carbonate accumulation. Although their net carbon balance remains uncertain due to the interplay between photosynthesis and calcification, recent studies suggest they can exhibit high uptake rates, warranting further investigation into their role in blue carbon assessments^38,39^.

In conclusion, the kelp forests of Northern Portugal represent a significant blue carbon stock, reinforcing their potential for inclusion in blue carbon management frameworks. Conservation and restoration of these forests are crucial for maintaining their ecological and economic value, including their blue carbon potential^9,11,12^. Like many other blue carbon ecosystems, kelp forests are vulnerable to climate change and human activities, making proactive management and potential restoration efforts essential to mitigate CO₂ emissions and enhance carbon sequestration. Climate change may further affect both the areal extent and biomass of kelp forests by altering ocean temperature, nutrient availability, and storm frequency, with possible implications for their blue carbon capacity.

To ensure effective conservation, integrating field data with large-scale models is critical for improving accuracy and refining strategies. Future research should focus on harmonizing methodological approaches, expanding monitoring efforts, and exploring regional variability to provide a comprehensive understanding of seaweed-based carbon fluxes. Strengthening these efforts will enhance the role of kelp forests in climate change mitigation and further emphasize their importance as a key component of blue carbon ecosystems.

## Electronic supplementary material

Below is the link to the electronic supplementary material.

Supplementary Material 1

## Data Availability

Data is provided within the manuscript or supplementary information files.
